# Gallbladder Wall Thickening associated with Dengue Shock Syndrome in a German traveller – no indication for surgical therapy – a case report

**DOI:** 10.1186/s40794-021-00148-0

**Published:** 2021-08-03

**Authors:** Noemi F. Freise, Björn Jensen, Verena Keitel, Tom Luedde

**Affiliations:** Department of Gastroenterology, Hepatology and Infectious Diseases, Duesseldorf University Hospital, Heinrich-Heine-University, Moorenstr. 5, 40225 Duesseldorf, Germany

**Keywords:** Dengue shock syndrome, Gallbladder Wall thickening, Acalculous cholecystitis, Capillary leakage

## Abstract

**Background:**

With the increasing number of dengue virus infections imported into Germany, knowledge about the different phases of the disease and possible complications is essential for the treatment of patients. The virus is endemic in the tropics and subtropics and up to 2.5 billion people are at risk of infection.

**Case presentation:**

Here we present a German traveller with dengue shock syndrome after returning from Thailand. After hospitalization the patient developed acute upper abdominal pain. The ultrasound findings were consistent with an acute acalculous cholecystitis, but were interpreted as dengue associated gallbladder wall thickening (GBWT). Therefore a surgical intervention was not indicated and would have been associated with an higher risk of complications in this situation. Under supportive care spontaneous regression of GBWT could be documented by sonography four days later as well as complete resolution of clinical symptoms.

**Conclusion:**

GBWT in dengue virus infection mimicking acute cholecystitis is a differential diagnosis one should take into consideration in travellers returning from endemic areas and should be managed conservatively because of an high risk of bleeding and increased mortality under surgical therapy.

## Background

Dengue fever is a viral infection with increasing incidence worldwide, almost 30-fold in the last 50 years. About 2.5 billion people are at risk of infection and 390 million infections are estimated to occur annually in 125 countries [1]. Dengue virus was isolated in 1943 in Japan (DENV 1) and 1945 in Hawaii (DENV 2) for the first time as a member of the family of *Flaviviridae*. Since then, several dengue epidemics have been described in the tropics and subtropics, as well as the spread of the virus through its main vector, *Aedes aegypti*, due to urbanization and globalization [2]. Until now there are 5 existing serotypes, DENV 1 to 4 and, discovered 2013, the DENV 5 [3]. Principal vectors of transmission are the female mosquitoes *A. aegypti* and *albopticus* which need a warm and humid climate and waterholes for breeding. Caused by climate warming and urbanization with availability of new artificial breeding sites made by humans, the natural habitat of the mosquitoes enlarges and therewith the virus spread out. The virus is prevalent in almost all tropical and subtropical regions of Asia, Africa, Australia, South Pacific, the Americas and some parts of the Middle East [4]. It is becoming a growing global health concern with high social and economic impact due to its rapid geographical extension, the increasing number of cases and disease severity [5].

Clinical symptoms in dengue fever manifest acutely after an incubation period of 4 to 7 days and can be divided into three phases: febrile, critical, and recovery. The febrile phase is marked by high-grade fever accompanied by myalgia and arthralgia, headache, often retro-orbital, nausea and vomiting. Mild hemorrhagic manifestations may occur as mucosal bleeding, petechias or gastrointestinal or vaginal bleeding (in women of reproductive age). Defervescence marks the following critical phase, usually 3–7 days after beginning of illness, typical symptoms as a macular rash and signs of capillary leakage may appear. At this point of illness, life-threatening symptoms must be recognized, for instance rising haematocrit, pleural effusion and ascites as signs of plasma leakage. At this stage shock can occur due to plasma leakage while in this phase the total white blood cell count and platelet count reach the lowest values. Since the risk of circulatory shock is high, a treatment with crystalloid fluid is the most valuable therapy [6]. In the following recovery phase, the leucocytes begin to increase, the platelets recover delayed, but after excessive fluid substitution respiratory distress can emerge [7, 8].

Studies from the high prevalence countries India and Taiwan showed that GBWT and signs of acalculous cholecystitis are not uncommon in patients with severe dengue [9, 10]. Reports of fulminant bleeding after surgery have also been reported in the literature [9, 11].

Here, we present a 23-year-old woman with ultrasound findings of marked acute cholecystitis associated with dengue fever after her return to Germany from Thailand and discuss the challenges of managing this condition in a low-incidence country.

## Case presentation

A healthy 23-year old woman has been travelling through Thailand for 4 weeks, also trekking in rainforest areas and she has been bitten by mosquitoes often. Two days after returning to Germany the patient presented in the emergency room of the University Hospital of Duesseldorf because of fever (39,2 °C) since 2 days, headache, myalgia and watery, not bloody diarrhoea. The clinical examination showed a maculopapular exanthema at extensor sites of arms and chest. The blood pressure was 111/66 mmHg, heart frequency 71/min, peripheral oxygen saturation 98% and respiratory rate 18/min, body temperature 39,2°Celcius. The chest-X-ray did not show any pathological result. Laboratory parameters showed leukopenia of 2.600/μl (4.0–11.0/μl), elevated c-reactive protein of 8,2 mg/dl (< 0,5 mg/dl) and Aspartat-Aminotransferase of 47 U/l (< 31.0 U/l), the international normalised ratio (INR) was lightly increased to 1.2 (0.9–1.1).

A Dengue rapid test (SD BIOLINE Dengue Duo) was negative for IgG and IgM, but positive for NS1-Antigen, tests of Malaria and other bacteria were negative. No further virologic tests were performed because the rapid test for dengue was already positive and the history and clinical presentation were consistent.

The following days the patient presented with low blood pressure under 100 mmHg systolic and under 50 mmHg diastolic with increasing haematocrit despite of daily administration of up to 4 l of crystalloids intravenously. Thus, according to the new WHO definition, the criteria for a Dengue Shock Syndrome (DSS) were fulfilled. The heart rate was still normal and only in the evening did the temperature rise slightly to a maximum of 38 °C. Two days later, the watery diarrhea stopped, but the nausea remained.

After 3 days of hospitalization the leukopenia decreased to 1.900/μl (4.0–11.0/μl) and for the first time a thrombocytopenia was detectable with a nadir of 29.000/μl (150-400 × 1000/μl) on day 6 after beginning of the symptoms, clinically no bleeding manifestations were present. At this time the patient complained of severe upper abdominal pain. Blood tests revealed elevated liver enzymes, Aspartat-Aminotransferase 106 U/l (< 31.0 U/l), Alanin-Aminotransferase 47 U/l (< 35.0 U/l) and gamma-GT 87 U/l (< 38.0 U/l) while the C-reactive Protein declined to an almost normal value and there was no abnormal bilirubin. Fever did not recur. Also, the lactate dehydrogenase increased significantly to 459 U/l (< 247 U/l) and we detected a rising haematocrit up to 45.4% (37.0–45.0%). The abdominal sonography showed a thickened gallbladder wall up to 21 mm with a reticular pattern, also free fluid in the gallbladder base and a punctual pressure pain. The spleen was only marginally increased with 12,4 cm, the liver was normal sized. There was a small pleural effusion on either sides but no ascites or further pathological findings in sonography - especially no cholestasis, no gallstones, and no hepatomegaly.

**Fig. 1:**
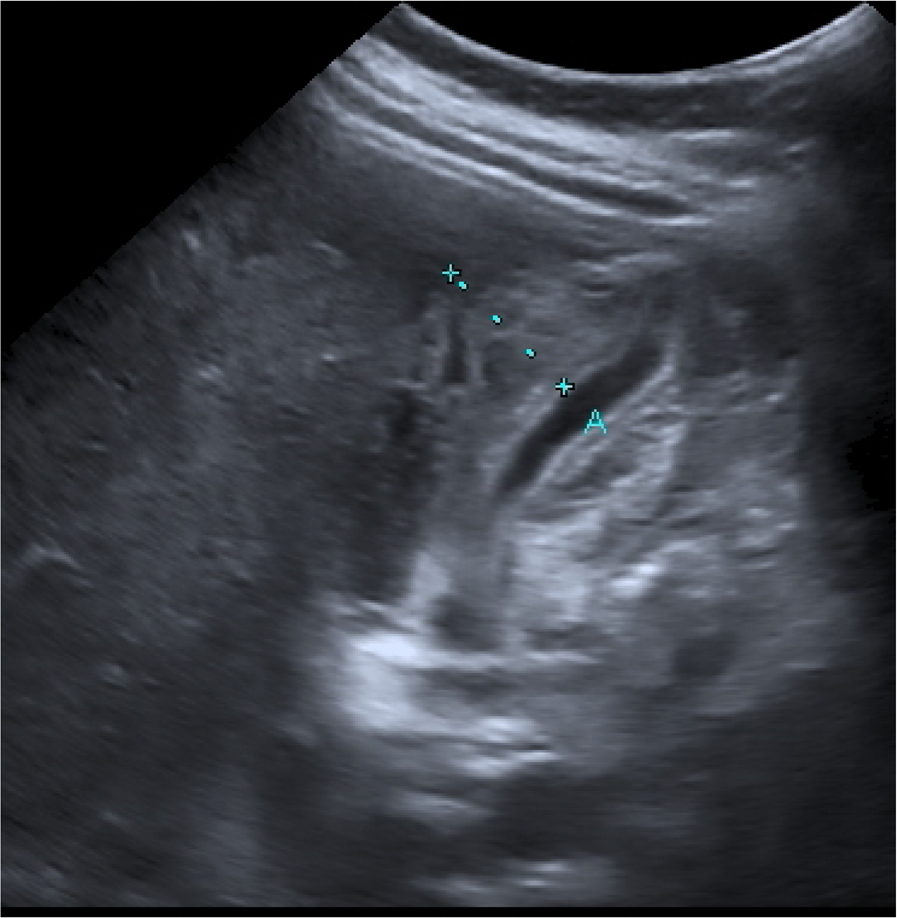
Transabdominal Ultrasound of the Gallbladder at the onset of abdominal pain. Distance A: 21 mm. Toshiba Aplio 500

**Fig. 2:**
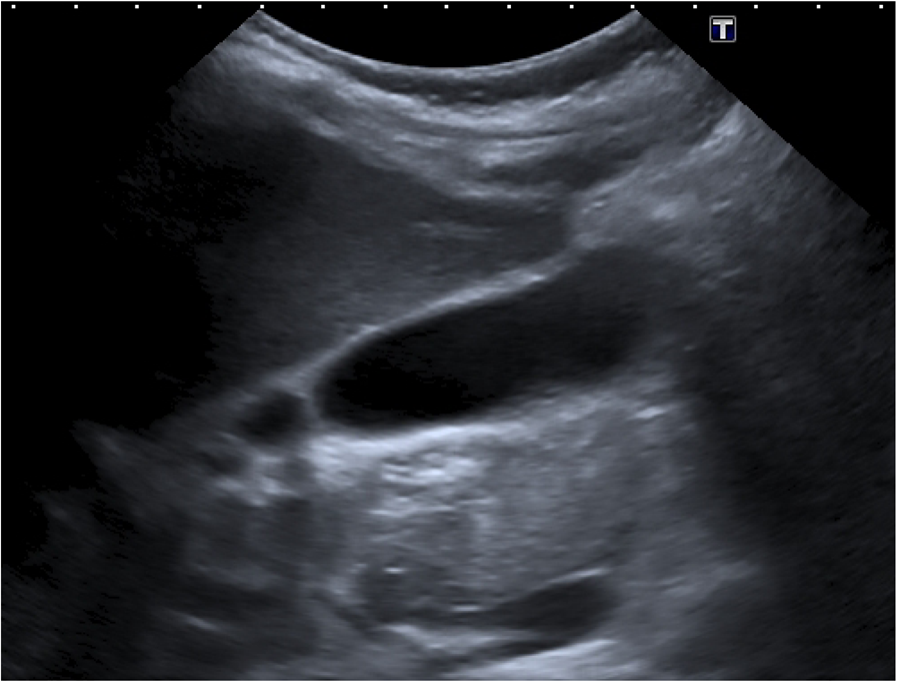
Transabdominal Ultrasound of the Gallbladder after regression of clinical symptoms. Toshiba Aplio 500

Because of classical morphological signs in sonography (Fig. 1) and the clinical presentation of an acute cholecystitis we were discussing the further treatment also with the surgical department, especially whether a surgical therapy is indicated. Because the patient was clinically stable with a decrease in C-reactive protein and no elevated bilirubin or cholestasis, we initiated antibiotic therapy only with ceftriaxone 2 g intravenously daily. We decided against surgical therapy, assuming that the ultrasound image of acute cholecystitis should be interpreted as GBWT due to dengue virus infection. Daily sonographies showed a rapid regression of the GBWT, the free fluid in the gallbladder base and the abdominal pain was not detectable anymore after 4 days (Fig. 2).

Seven days after hospitalization the general condition of the patient improved, there was no increased temperature in the last 4 days. Leucocytes and thrombocytes counts increased slowly and liver enzymes continued to decrease. Therefore we stopped the intravenous fluid substitution and the antibiotics, 2 days later the patient could be discharged from hospital.

Blood examinations 8 days after discharge and 20 days after beginning of the symptoms showed a positive serology for Dengue: Dengue-IgG was higher than 200.00 RU/ml (< 22 RU/ml) and the Index to Dengue-IgM was 2,12 (Index >/= 1.1), confirming Dengue-Infection. Leucocytes, thrombocytes, liver enzymes and the lactate dehydrogenase were in a normal range. Clinically, the patient was in good general condition, and the exanthema had completely disappeared.

## Discussion

While dengue fever causes approximately 390 million infections and nearly 25,000 deaths worldwide annually, only 956 imported cases were reported to the Robert Koch Institute in Germany in 2016 [12, 13]. Although this was the highest number reported to date, and thus dengue fever is gaining importance as a differential diagnosis in travellers returning from endemic areas, it is still a rare diagnosis in Germany.

The new WHO Classification of Dengue cases differentiates between Dengue with or without warning signs and severe Dengue including Dengue Shock Syndrome, haemorrhagic fever and organ impairment [7]. Our patient suffered from symptoms of a severe Dengue and a DSS but without any bleeding signs despite a low platelet count. Systolic blood pressure was under 100 mmHg at any time and the haematocrit was rising in defiance of a up to 4 l of Jonosteril® Ringer’s lactate daily intravenously. At the onset of upper abdominal pain, 3 days after admission, sonographic evidence of gallbladder wall thickening (GBWT) of 21 mm with a reticular pattern and signs of capillary leak syndrome as pleural effusion and peripheral oedema was found in a young, and otherwise healthy patient. This is typical of plasma leakage and may be considered an indicator of DSS [14]. At this point the parameters of inflammation as C-reactive protein and liver enzymes were already decreasing. An elevated direct bilirubin was never detected as well as there was no cholestasis in sonography. Thus, we assumed a GBWT due to Dengue infection which is well described. A study from India showed that 14 of 27 adult patients with Dengue fever and abdominal pain presented signs of an acalculous cholecystitis, defined by a gallbladder wall thicker than 3 mm, positive Murphy’s sign and a striated gallbladder [11]. Khor et al. noticed cholecystitis in 7.6% of the patients, 4% had with Dengue Haemorrhagic Fever (DHF), of which 3.05% had acute cholecystitis (acalculous 1.82%, calculous 1.22%), hardly any deviating data found Wu et al. in his study from Taiwan with 131 patients with Dengue fever; acute acalculous cholecystitis in 7.63% of patients with dengue fever [15,9]. Large multicentre analyses regarding mortality and morbidity of acute acalculous cholecystitis in dengue fever are not available. The results of the studies by Shamim et al., Wu et al. and Khor et al. were both able to show that surgical interventions lead to a prolonged hospital stay, an higher need for blood transfusions and wound healing problems [9, 15, 16]. In the study by Shamim, et al. mortality in patients with acute abdomen was 4.65%, while there was no mortality in patients without acute abdomen [16]. In 2009 a German female 21-year-old traveller with Dengue fever with signs of an acalculous cholecystitis underwent surgery and subsequently died because of massive bleeding of the abdominal wound due to thrombocytopenia and coagulation dysfunction [11]. Another patient underwent appendectomy because of massive abdominal pain, the explorative laparotomy showed a normal appendix [17]. Taking into account these publications we decided against surgical therapy because we assumed GBWT caused by plasma leakage due to the dengue shock syndrome and not by an infectious or calculous cholecystitis. Antibiotic therapy has been started though with ceftriaxone 2 g daily intravenously. Three days later the symptoms resolved and 4 days later there was no more GBWT detectable by ultrasound.

Twenty days after the beginning of the symptoms we could verify the dengue infection by a positive serology with a high IgM and IgG. The rapid test of non-structural protein 1 (NS1, SD BIOLINE Dengue Duo) had been positive at the second day after onset of symptoms, this test is valuated to be highly specific [18]. Specific dengue IgM-antibodies are usually detectable at least 4 days after beginning of fever [19]. Liver enzymes, leucocytes and thrombocytes were in a normal range at this point. The patient could be released soon and recovered fully. Unfortunately, the serotype was not determined, but it would have been interesting for the assessment of the clinical case. Vaughn et al. found that infection by DENV-2 caused more severe courses of disease in patients hospitalised for dengue fever, possibly due to the greater viral replication of this serotype [20]. Very frequent clinical checks of the patient’s volume status were performed, but detailed documentation of the fluid balance was unfortunately not done and is another limitation of this case report. Formal assessment of fluid balance is advisable to better evaluate the progression of DSS. Since severe courses of dengue fever are rare in Germany, this case report shows that complications also occur in young and healthy travellers and that we should consider such courses in differential diagnostic considerations.

## Conclusion

Dengue fever in Germany is still a rather rare diagnosis, but due to travel activities to endemic areas not a disease to be forgotten. Given the uncertainty that often exists in dealing with the disease in Germany and the risk of developing Dengue Shock Syndrome, a well thought-out procedure is necessary. In conclusion of this case presentation, signs of an acute abdominal pain in combination with the ultrasound finding of acalculous cholecystitis should not be treated surgically if a Dengue infection is assumed or proven. Conservative management under continuous observation is advised to prevent unnecessary morbidity and mortality in patients with dengue fever and GBWT.

## Data Availability

Not applicable.

## Abbreviations

GBWT: Gall Bladder Wall Thickening
WHO: World Health Organization
DHF: Dengue Haemorrhagic Fever
DSS: Dengue Shock Syndrome
